# Evaluating COVID‑19 Risk to Essential Workers by Occupational Group: A Case Study in Massachusetts

**DOI:** 10.1007/s10900-023-01249-x

**Published:** 2023-07-29

**Authors:** Beth M. Haley, Prasad Patil, Jonathan I. Levy, Keith R. Spangler, Koen F. Tieskens, Fei Carnes, Xiaojing Peng, R. Monina Klevens, T. Scott Troppy, M. Patricia Fabian, Kevin J. Lane, Jessica H. Leibler

**Affiliations:** 1 Department of Environmental Health, School of Public Health, Boston University, 715 Albany St, Boston, MA 02118, USA; 2 Department of Biostatistics, Boston University School of Public Health, Boston, MA, USA; 3 Bureau of Infectious Disease and Laboratory Sciences, Massachusetts Department of Public Health, Boston, MA, USA

**Keywords:** COVID-19, Essential workers, Geospatial data, Community health, Occupational health

## Abstract

Occupational exposure to SARS-CoV-2 varies by profession, but “essential workers” are often considered in aggregate in COVID-19 models. This aggregation complicates efforts to understand risks to specific types of workers or industries and target interventions, specifically towards non-healthcare workers. We used census tract-resolution American Community Survey data to develop novel essential worker categories among the occupations designated as COVID-19 Essential Services in Massachusetts. Census tract-resolution COVID-19 cases and deaths were provided by the Massachusetts Department of Public Health. We evaluated the association between essential worker categories and cases and deaths over two phases of the pandemic from March 2020 to February 2021 using adjusted mixed-effects negative binomial regression, controlling for other sociodemographic risk factors. We observed elevated COVID-19 case incidence in census tracts in the highest tertile of workers in construction/transportation/buildings maintenance (Phase 1: IRR 1.32 [95% CI 1.22, 1.42]; Phase 2: IRR: 1.19 [1.13, 1.25]), production (Phase 1: IRR: 1.23 [1.15, 1.33]; Phase 2: 1.18 [1.12, 1.24]), and public-facing sales and services occupations (Phase 1: IRR: 1.14 [1.07, 1.21]; Phase 2: IRR: 1.10 [1.06, 1.15]). We found reduced case incidence associated with greater percentage of essential workers able to work from home (Phase 1: IRR: 0.85 [0.78, 0.94]; Phase 2: IRR: 0.83 [0.77, 0.88]). Similar trends exist in the associations between essential worker categories and deaths, though attenuated. Estimating industry-specific risk for essential workers is important in targeting interventions for COVID-19 and other diseases and our categories provide a reproducible and straightforward way to support such efforts.

## Background

The risk of exposure to SARS-CoV-2—the virus that causes COVID-19—has varied widely among workers, especially during the first year of the pandemic when policy measures necessitated shifting many jobs to working from home [1]. However, workplaces that served essential functions for communities, such as healthcare, food production, and public safety, remained open with varied mitigation strategies [2]. Those working in person have generally experienced greater COVID-19 disease burden than those working from home [3–5], but even among in-person workers, increased occupational risk has varied given workplace differences in contact with infected individuals, the public, and co-workers; levels of indoor ventilation; and worksite interventions [3, 6–9]. Early in the pandemic, elevated risk of infection was well-documented among some worksites including healthcare settings [10], food processing centers [11], food service locations [12], and transportation networks [13], with retrospective analyses also identifying elevated risk for workers in material moving, production, building construction and maintenance, and protective and social services [4, 5, 14–18].

Occupation data are not consistently collected during disease surveillance activities, including for COVID-19 [19], so there remain gaps in understanding which workers experienced elevated risk, especially of COVID-19 infection (as opposed to hospitalization or death). Some analyses have considered essential workers as a homogenous group [20–22], while others have found differences in specific occupational risk factors for mortality [14, 16, 17] and hospitalization [17, 18]. Improved understanding of the differences in risk of COVID-19 infection among essential worker occupations can inform more targeted public health interventions in occupational settings, and can potentially reduce health disparities given the overrepresentation of African American, Latinx, and Native American workers within essential occupations [1, 7, 9, 23, 24].

In this study, we use publicly available occupation data to develop a novel set of essential worker categories among the occupations designated as COVID-19 Essential Services in Massachusetts (MA) by the Governor’s emergency order in March of 2020 [25]. We describe the spatial distribution of these essential worker groups, both statewide and among towns that experienced high COVID-19 burden in the first year of the pandemic (March 2020 to February 2021). We assess the relationship between COVID-19 cases and deaths and our new essential worker categories at census tract resolution, controlling for sociodemographic predictors of outcomes. We evaluate the correlation between metrics of community-level mobility and essential worker categories to account for the dynamic nature of stay-at-home restrictions and in-person work over the time period of the study [26]. We also test interaction terms between essential worker categories and race/ethnicity at the tract level, given evidence elsewhere of differential risk by race/ethnicity within occupational category [14–16].

## Methods

### Data Sources

#### Occupation Data

We accessed estimates of the number of working adults per census tract by occupation from the American Community Survey (ACS) five-year estimates (2015–2019) [27]. ACS occupation data are categorized according to the 23 major occupational groups identified by the 2018 Standard Occupational Classification (SOC) System [28].

#### COVID‑19 Outcomes

Individual-level COVID-19 case and death data from March 1, 2020 through February 13, 2021 were obtained from the Massachusetts Department of Public Health (MDPH) under a unique data use agreement. Cases and deaths within the dataset were confirmed by established laboratories via nucleic acid amplification tests. Date of diagnosis or death and residential address were included for each individual. As described in previous work [21], residential addresses were geocoded and used to attribute case or death to the census tract of the associated residence. MA census tract (2010), town, and county boundaries were obtained from MassGIS [29]. Institutional residences, such as long-term care facilities and homeless shelters, were also identified in the geocoded address dataset. To focus this analysis on disease patterns within the general community, cases and deaths among people living in institutional residences were removed from the outcome dataset, as detailed in our prior work [21].

#### Time Period

We aggregated daily cases and deaths to two time periods corresponding to the first two waves of the pandemic: Phase 1 (March 1 to June 6, 2020) and Phase 2 (September 13, 2020 to February 13, 2021). Given that there were relatively few COVID-19 cases and deaths during the summer of 2020, we excluded this time period from our analysis.

#### Sociodemographic Covariates

We extracted census tract-level covariates from the ACS: percent of the population that identifies as Black or African American (% Black), Hispanic or Latino (% Latinx), or American Indian/Alaska Native (% AIAN); percent of the population younger than 20 years old (% Age < 20) or over 80 years old (% Age > 80); percent of residents enrolled as undergraduate students (% Undergrad); percent of the population without health insurance (% Uninsured), living in poverty (% Under federal poverty line), or living in conditions with more than 1.5 people per room (% Crowding); the household median income (HMI); and housing unit density (HUD).

#### Mobility data

We estimated population mobility by tract to incorporate data on stay-at-home restrictions and population movement into our models. Estimates of mobility were calculated from the SafeGraph Social Distancing Metrics (SDM) dataset [30]. SDM data are derived from anonymized smartphone devices whose users have opted in to location sharing. In aggregate, the devices in the SafeGraph dataset represent about 10% of the devices in the United States [31]. We used the following mobility metrics, aggregated to the census tract-level in MA with population weighting, averaged over the time period of each phase and evaluated continuously [20, 26]: percent of devices that remained entirely at home; part-time work behavior (spending 3–6 h at a location other than home between 8 am and 6 pm); full-time work behavior (spending more than 6 h at a location other than home between 8 am and 6 pm); and any work behavior (sum of part- and full-time work behavior).

### Essential Worker Categories

Using the MA definition of essential services [32] that was established in the spring of 2020 by the Governor’s executive order, we matched essential occupations to the SOC major groups within the ACS dataset. From the list of selected SOC major groups, we then combined occupational groups into larger categories based on similarity of the type of work represented in those groups (e.g., construction and building maintenance), contact with members of the public (e.g., in-person services), and the potential for elevated exposure to SARS-CoV-2 (e.g., healthcare settings). Categories were established in conversation and consensus among our team, which includes environmental and occupational health researchers. We included all SOC groups that contained any designated essential service in the state definition, except for “Education Instruction and Library Occupations” and “Farming, Fishing, and Forestry Occupations”, as the former involved largely remote engagement early in the pandemic and the latter has few workers in MA. This process resulted in the following five essential worker categories: 1) Construction/Transportation; 2) Production; 3) Public-facing workers; 4) Healthcare; and 5) Limited exposure workers (Table 1).

### Analytical Methods

To visualize the data, we mapped the novel essential worker categories as percentage of total population at the census tract-level, both statewide and with a focus on five MA communities with high COVID-19 case incidence over the time period of this study [33]: Chelsea, Everett, Lawrence, Lynn, and Revere.

We modeled non-institutional cases and deaths at the census tract-level, each over the two phases, calculating incidence rate ratios (IRR) and 95% confidence intervals (CI) for all predictors using mixed-effects negative binomial regression models (four models total), following Spangler et al. [21] Census tract population was used as an offset term to account for differences in population among census tracts. Predictors were included in the model based on previous findings [20, 21] as well as relevance for health disparities, COVID-19 transmission, and/or COVID-19 disease severity [34, 35]. All continuous predictors were standardized to zero mean with standard deviation of 1. Essential worker categories were included in the model as categorical variables to simplify interpretation of interactions and other results; categories “high”, “medium”, and “low” correspond to the tertiles of the percent of tract population represented by each category, with “low” as the reference category. The county in which the census tract is located (of 14 counties in MA) was included as a random effect to control for spatial autocorrelation of residuals for tracts in the same region of the state. Interaction terms between the essential worker categories and the proportions of Black and Latinx residents were also included in the model to investigate possible interactions between occupation and race/ethnicity. Associations for which the 95% confidence interval excludes 1.0, the null value, were considered statistically significant. We calculated Pearson correlation coefficients between all mobility metrics and essential worker categories (as continuous percentage of tract population) in an exploratory analysis to determine the degree to which mobility may be associated with the relationship between occupation and COVID-19. Mixed effect modeling was executed with the *glmmTMB* function from the *glmmTMB* package (version 1.1.2.3) [36] in R (version 4.1.2) [37].

## Results

### Essential Worker Categories

**Fig. 1:**
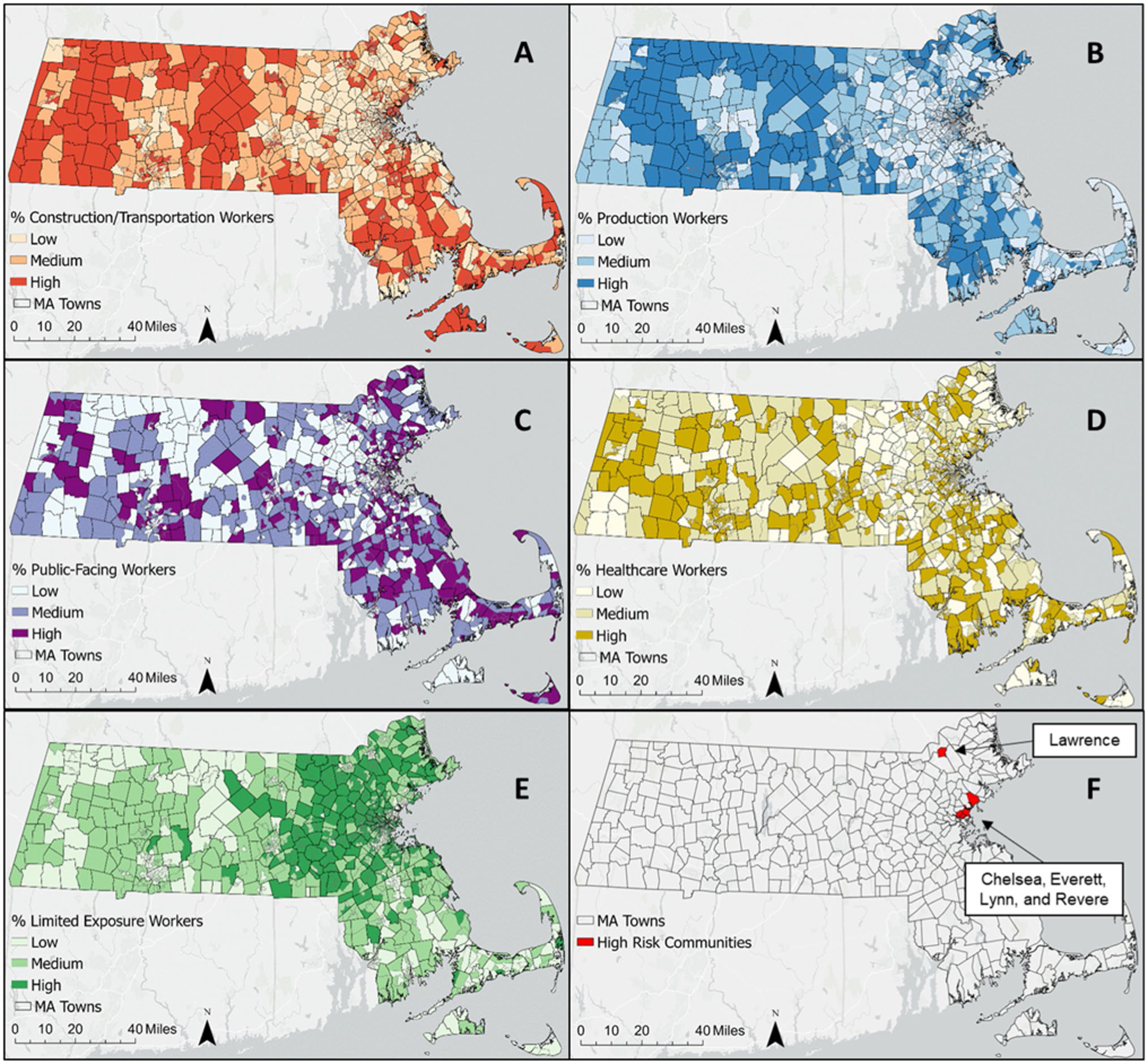
Spatial distribution of essential workers categories at the census tract level and high risk communities. Essential worker categories are as follows: **A** Construction/Transportation workers; **B** Production workers; **C** Public-facing workers; **D** Healthcare workers; **E** Limited Exposure workers. **F** Shows the location of five MA communities with high COVID-19 case incidence over the study period. Worker populations are depicted as tertiles

Figure 1 shows the statewide distribution of essential worker categories at the census tract-level. The Construction/Transportation and Production worker categories follow similar patterns, with the majority of high percentile census tracts located in the western, central and southeastern regions of the state (Figs. 1a and 1b). The distributions of Public-facing and Healthcare workers (Figs. 1c and 1d) have a higher degree of spatial heterogeneity without a clear pattern by region. The statewide distribution of Limited Exposure workers (Fig. 1e) is markedly different from the other essential worker categories, with the majority of high percentile census tracts located in eastern and northern MA in the Boston metro area.

**Fig. 2:**
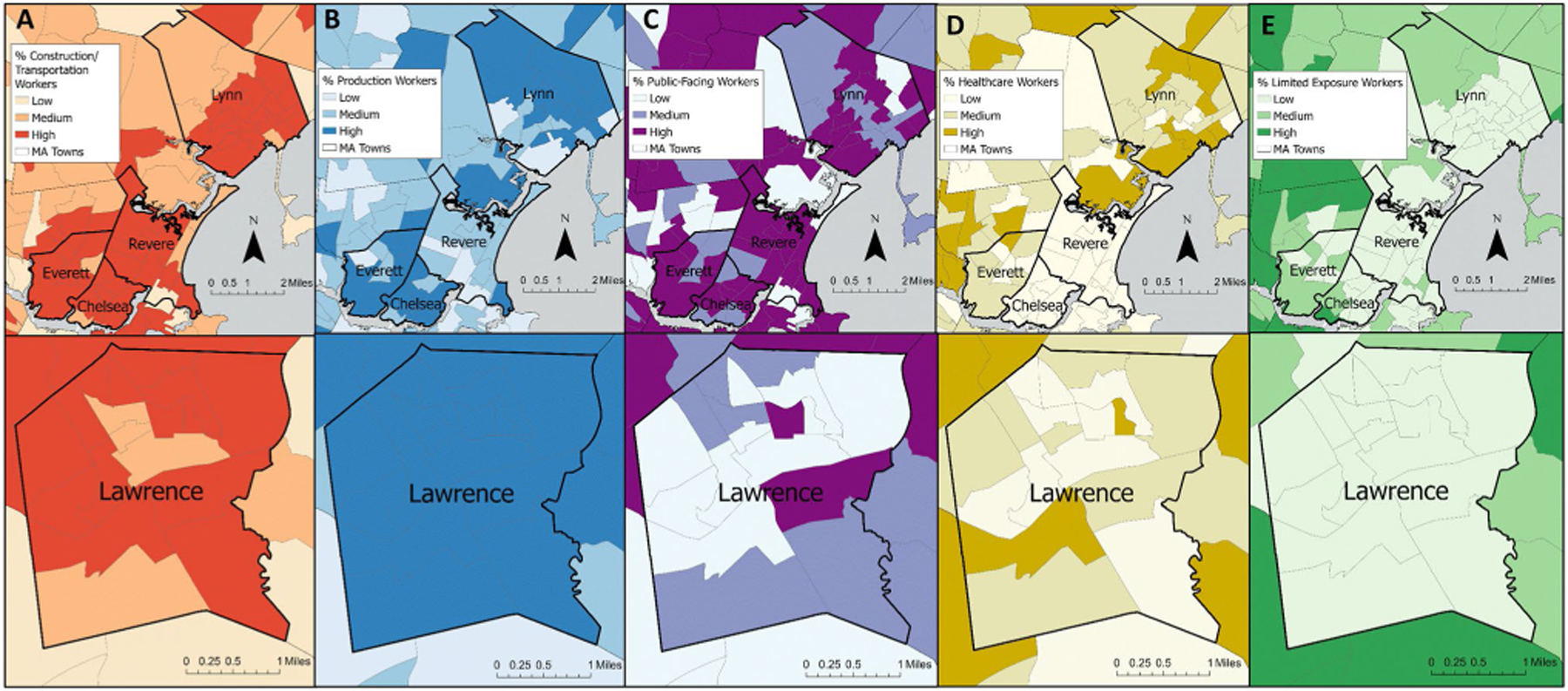
Essential worker distribution at the census tract level in five Massachusetts cities. Chelsea, Everett, Lynn and Revere shown in the top row with Lawrence shown in the bottom row. Essential worker categories are as follows: **A** Construction/Transportation workers; **B** Production workers; **C** Public-facing workers; **D** Healthcare workers; **E** Limited Exposure workers. Worker populations are depicted in tertiles

Examining the spatial distribution of essential workers within five high-risk communities (Fig. 2), most tracts were in the highest percentile for Construction/Transportation workers (9.8–31.0% of total tract population) as well as Production (2.7–12.4%) and Public-Facing workers (12.8–26.8%) but in the lowest percentile for Limited Exposure workers (less than 5.3%), though with some variation within and between communities.

### COVID‑19 Outcomes and Exploratory Analysis of Mobility Data

In total, Phase 2 included about five times the non-institutional cases reported in Phase 1 (393,541 vs. 79,349, respectively) and approximately 25% more non-institutional deaths (3535 vs. 2696, respectively). Details regarding case and death burden in MA as well as sociodemographic detail of census tracts included in the analysis are reported in Table S1.

The results of our exploratory analysis of mobility metrics are shown in the correlation matrix in Figure S1. Because none of the mobility metrics investigated were strongly correlated (|*r*|> 0.60) with the percent of essential worker categories among census tracts (continuous variable), mobility metrics were not included in the regression analyses.

### Regression Analyses

**Fig. 3:**
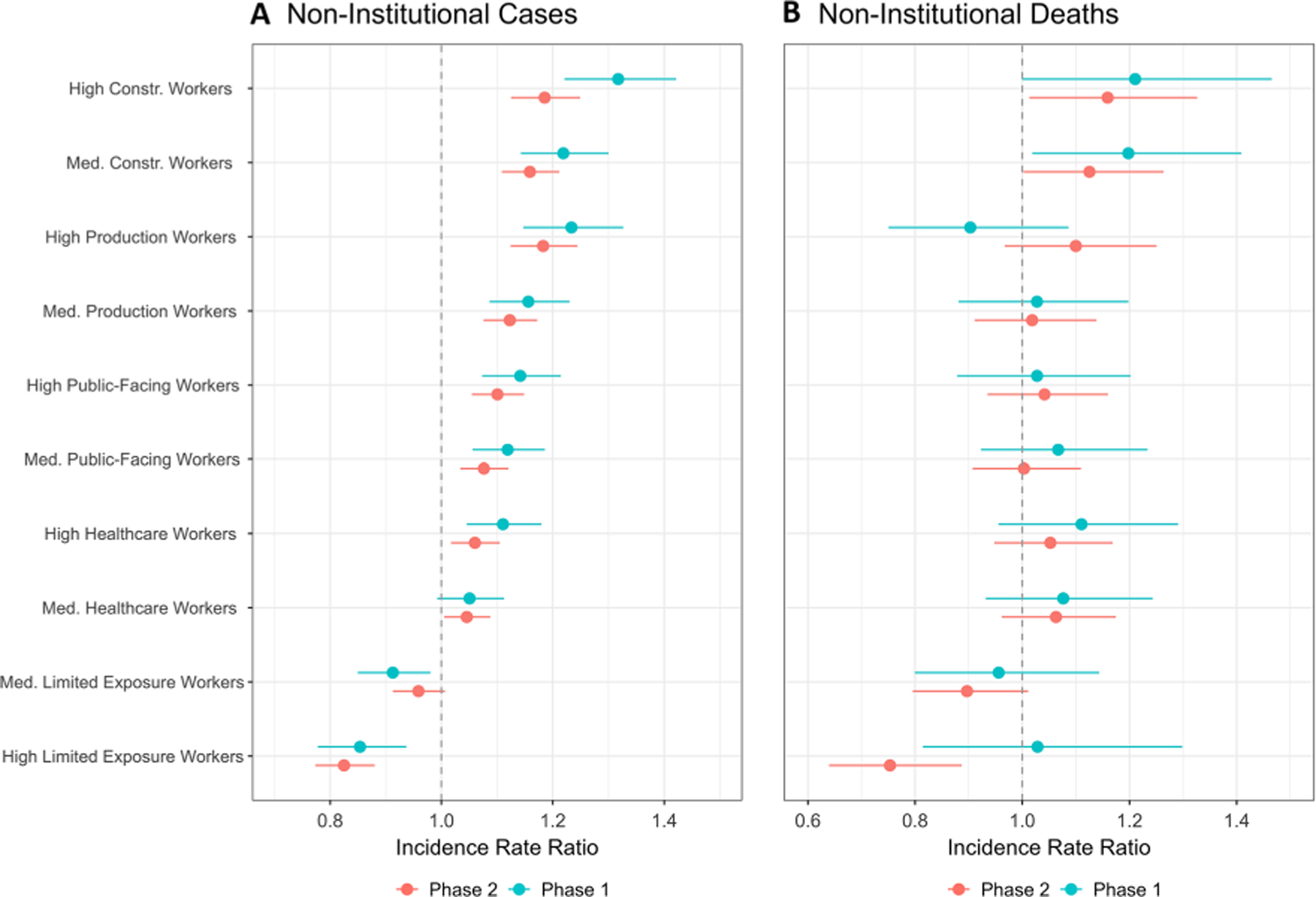
Incidence rate ratios (IRR) and 95% confidence intervals for census tracts with high and medium percentiles of essential workers. **A** Reflects non-institutional cases and **B** non-institutional deaths. Data is presented in two phases of the pandemic: Phase 1: March–June 2020 in blue, top; Phase 2: September 2020–February 2021 in red, bottom. The overall incidence rate of all non-institutional cases was 1.16% in Phase 1 and 5.75% in Phase 2. The overall incidence rate of all non-institutional deaths was 0.04% in Phase 1 and 0.05% in Phase 2. Incidence rates for all occupational and percentile groups are summarized in Table S3

Census tracts with high and medium percentiles of Construction/Transportation, Production, Public Facing, and Healthcare essential workers had elevated incidence of community cases compared to census tracts with low percentiles of workers in those categories (Fig. 3; Table S2).

Tracts with high percentiles of Construction/Transportation and Production workers experienced the largest increases in case incidence (IRR 1.32 [95% CI 1.22, 1.42] and 1.23 [1.15, 1.33] in Phase 1, respectively). Conversely, census tracts with high and medium percentiles of Limited Exposure essential workers had lower incidence for community cases than those with low percentiles of Limited Exposure essential workers (e.g., High, Phase 1: 0.85 [0.78, 0.94]; Phase 2: 0.83 [0.77, 0.88]. Associations between essential worker categories and case incidence were statistically significant during both Phases with the exception of census tracts with medium percentiles of Healthcare workers in Phase 1 (1.05 [0.99, 1.11]) and tracts with medium percentiles of Limited Exposure workers in Phase 2 (0.96 [0.91, 1.01]). The directionality of the associations between essential worker categories and community cases remained stable for all categories between Phase 1 and Phase 2, and the magnitude of the association was smaller in Phase 2 for all categories except for census tracts with high percentiles of Limited Exposure workers.

For non-institutional deaths, estimates are more imprecise given the smaller number of COVID-19 deaths compared to cases, but the patterns are similar (Fig. 3, Table S2). Census tracts with high and medium percentiles of essential workers in the Construction/Transportation, Public-Facing, and Healthcare categories had higher incidence of death compared to census tracts with low percentiles of workers in those categories in both phases. Census tracts with high percentiles of Production workers had reduced incidence of death in Phase 1 (0.90 [0.75, 1.09]) and increased incidence of death in Phase 2 (1.10 [0.97, 1.25]). Census tracts with high percentiles of Limited Exposure workers had marginally elevated incidence of death in Phase 1 (1.03 [0.81, 1.30]) and significantly reduced incidence in Phase 2 (0.75 [0.64, 0.89]).

When testing interaction terms between the essential worker categories and the proportion of Black and Latinx residents, we found that a few were significantly associated with community cases or deaths (Table S4), but inclusion of these terms did not meaningfully affect the interpretation of associations between essential worker categories and community cases or deaths.

## Discussion

We found differential associations between various categories of essential workers and both COVID-19 case incidence and mortality in MA, highlighting potential occupational exposure risk that extends beyond a homogenous essential worker designation. Differential risk by occupational category persisted after controlling for tract-level characteristics related to race/ethnicity, age, income, and housing. This finding suggests that detailed employment patterns are important predictors of COVID-19 risk independent of sociodemographic factors.

Multiple factors may explain these patterns. Elevated COVID-19 case incidence for healthcare workers has been well-documented [10] and is not surprising given exposure risks associated with patient interactions. An analysis of workplace characteristics by occupation found higher exposure risk among workers in close contact with other people in indoor (i.e., healthcare, food preparation and serving, personal care and services, and educators) or outdoor spaces (i.e., protective services, building and grounds cleaning and maintenance, construction and extraction, and transportation and material moving workers) [7]. According to an analysis of occupational data that incorporated work-from-home feasibility and industries that were shut down during the first wave of the pandemic in the US, the highest percentages of in-person workers were in the following occupations: sales and related (14.8%), healthcare practitioners (14.1%), transportation and material moving (13.9%), construction and extraction (10.4%), production (10.3%), and food preparation and serving (10.2%) [1]. These patterns are consistent with the positive associations between Construction/Transportation, Production, Public Facing, and Healthcare workers and COVID-19 case incidence observed in our analysis. Our results and those of similar studies provide evidence that risk of COVID-19 infection, severe disease, and death vary drastically by worker occupation [4, 5, 14–18].

We observed high percentages of certain essential worker categories in five of the most COVID-19-impacted cities in the state of MA during the first year of the pandemic (Chelsea, Everett, Lawrence, Lynn and Revere). The majority of the census tracts in all of these cities were in the highest tertile for percent Construction/Transportation workers, Production workers, and/or Public-Facing workers. The presence of a greater percentage of essential workers in the occupational categories that are most strongly associated with COVID-19 cases in these communities compared to other areas of the state suggest that occupational exposure may have played a role in community transmission. However, given the high rates of pandemic-related unemployment in the first six months of the pandemic, it is possible that high percentages of essential workers in these industries in these communities indicate instead that elevated risk of COVID-19 was associated with higher rates of unemployment. In general, workers in the lowest wage quartile were most susceptible to unemployment; as of September 2020, employment rates among workers in the lowest wage quartile were 22.8% below pre-pandemic rates [38]. As a result, we cannot draw formal conclusions here using static employment categories derived before the pandemic. It is also important to note that the majority of census tracts in these five cities are environmental justice communities per the MA state definition [39], indicating that there may be additional risk factors at play not captured in our models. However, to the extent that occupational data proxy for factors related to pandemic-associated unemployment, our findings do reflect common experiences shared by communities with a greater proportion of in-person essential workers.

A key limitation to our analysis is that we lacked occupational data at the individual level. It is therefore possible that we have captured other attributes of census tracts that correlate with occupational patterns, such as high rates of pandemic-related unemployment as discussed above. Use of 2019 occupation data does not account for temporal or regional patterns of unemployment, furloughs, and/or industry-level shutdowns and re-openings that occurred during the study period. The SOC occupational delineations do not perfectly align with the state-level definitions, likely leading to some misclassification in the count of essential workers by census tract. The ACS may also undercount undocumented or migratory workers [40], which could particularly impact some occupations–such as construction and production–more than others. We found little evidence of interaction between race/ethnicity and essential worker categories in either the case or death models assessed in this study, possibly due to sample size issues. Finally, the mobility data used in the exploratory analysis may not be representative of the population at census tract resolution due to a relatively low percentage of mobile phones included [31]. There is no sociodemographic data available with the SafeGraph dataset, so the extent of selection bias within the dataset is unknown, potentially explaining the weak association between mobility and our essential worker categories.

Strengths of this study include the use of individual-level, molecular-confirmed case and death data from MDPH, which allowed us to isolate non-institutional outcomes at the census tract level for use in the regression analysis and focus solely on community-level risk factors and endpoints. Inclusion of cases and deaths over two periods during the first year of the pandemic allowed for a more refined analysis, in light of changes in public policies during this time. Finally, inclusion of multiple sociodemographic variables found to be important drivers of COVID-19 outcomes reduces potential confounding in our analyses by these factors, although the risk of residual confounding remains.

## Conclusions

Our findings indicate that census tracts with higher proportions of workers in construction, transportation, buildings maintenance, production, and public-facing sales and services occupations faced elevated risk of COVID-19 over the first year of the pandemic in MA. The occupational composition of census tracts may have played a role in COVID-19 transmission. Collection of occupational data alongside case data would improve efforts to identify and prioritize vulnerable communities and target interventions.

## Supplementary Material

Supplementary Material

## Figures and Tables

**Table 1:** Descriptive statistics of essential worker categories

Essential worker category	Standard Occupational Classification system major groups included	Statewide total number of workers	Median % across all census tracts	Tertile ranges (%)
All essential workers	(all below)	2,398,032	35.19	Low: 0–32.7 Medium: 32.8–37.2 High: 37.3–59.0
Construction/ Transportation	Construction and extraction occupations Building and grounds cleaning and maintenance occupations Transportation and material moving occupations Installation, maintenance, and repair occupations	543,412	7.96	Low: 0–6.1 Medium: 6.2–9.7 High: 9.8 – 31.0
Production	Production occupations	145,185	1.72	Low: 0–1.1 Medium: 1.2–2.6 High: 2.7–12.4
Public-Facing	Sales and related occupations Food preparation and serving related occupations Personal care and service occupations Community and social service occupations Protective service occupations	795,169	11.52	Low: 0—10.3 Medium: 10.4–12.7 High: 12.8–26.8
Healthcare	Healthcare practitioners and technical occupations Healthcare support occupations	367,550	5.23	Low: 0–4.5 Medium: 4.6–6.1 High: 6.2–19.3
Limited Exposure	Business and financial operations occupations Computer and mathematical occupations Architecture and engineering occupations Arts, design, entertainment, sports, and media occupations	546,716	7.04	Low: 0–5.3 Medium: 5.4–9.1 High: 9.2–41.5

Occupation data was accessed via the American Community Survey five-year estimates (2015–2019) [27]. Essential worker categories were developed based on the Massachusetts Essential Services definition [32], as described in the text

## Data Availability

The COVID-19 outcome data were made available to the authors by the Massachusetts Department of Public Health under a data use agreement and cannot be shared by the authors. Select mobility data products are available to academic researchers from SafeGraph. The tract-level occupation and covariate data from the American Community Survey and other public sources are available from the corresponding author upon reasonable request.
