# Supplementary material for: Evaluating COVID‑19 Risk to Essential Workers by Occupational Group: A Case Study in Massachusetts
Source: J Community Health. Author manuscript; Available in PMC 2024 Mar 14. (PMC10823035; doi:10.1007/s10900-023-01249-x)
Supplement: Supplementary Material [file NIHMS1940243-supplement-Supplementary_Material.pdf]

Evaluating COVID-19 risk to essential workers by occupational group: A case study in Massachusetts

Journal of Community Health

Beth M. Haley[1], Prasad Patil[2], Jonathan I. Levy[1], Keith R. Spangler[1], Koen F. Tieskens[1], Fei Carnes[1], Xiaojing Peng[2], R. Monina Klevens[3], T. Scott Troppy[3], M. Patricia Fabian[1], Kevin J. Lane[1], Jessica H. Leibler[1]

1. Department of Environmental Health, Boston University School of Public Health, Boston, MA, USA
2. Department of Biostatistics, Boston University School of Public Health, Boston, MA, USA
3. Bureau of Infectious Disease and Laboratory Sciences, Massachusetts Department of Public Health, Boston, MA, USA

Corresponding author:
Jessica H. Leibler
Department of Environmental Health
Boston University School of Public Health
715 Albany St.
Boston, MA, 02118
jleibler@bu.edu

SUPPLEMENTARY MATERIALS:

Table S1: Descriptive statistics of non-institutional COVID-19 cases and deaths, and average census tract characteristics in Massachusetts, USA[a].

	**Phase 1 (March 2020 – June 2020)**		**Phase 2 (September 2020 – February 2021)**	
	Cases	Deaths	Cases	Deaths
Number of individuals[b]	79,349	2696	393,541	3535
**Mean Tract-Level Characteristics**				
Variable Name	Description			Mean (Std. Dev.)
% Black	Percent of the population that identifies as Black or African American			7.94% (13.19%)
% Latinx	Percent of the population that identifies as Latino or Hispanic			12.66% (16.83%)
% AIAN	Percent of the population that identifies as American Indian or Alaska Native			0.23% (0.64%)
% Age < 20	Percent of the population younger than 20 years old			22.64% (6.73%)
% Age > 80	Percent of the population over 80 years old			4.23% (2.72%)
% Undergrad	Percent of the population enrolled as undergraduate students			6.48% (10.65%)
% Uninsured	Percent of the population without health insurance			2.47% (2.12%)
% Under federal poverty line	Percent of the population living in poverty			10.80% (9.84%)
% Crowding	Percent of the occupied units with more than 1.5 people per room			0.71% (1.23%)[c]
HMI	Household median income (USD)			$85,878.79 ($37,892.54)[d]
HUD	Housing unit density (units/mi^2)			3093.25 (5058.34)

[a] Only census tracts with population greater than 0 included in the analysis (n=1462).

[b] Reflects the number of cases and deaths that were successfully geocoded to a census tract (total number of individuals excluded due to lack of geocoding is 1360 [0.27%]).

[c] Three census tracts omitted because number of occupied units was 0.

[d] Twelve census tracts omitted because household median income data missing.

Figure S1: Correlation matrix of essential worker groups (percent of total tract population) and metrics of mobility from SafeGraph's Social Distancing Metrics dataset. Correlations between essential worker categories and mobility metrics circled for emphasis.

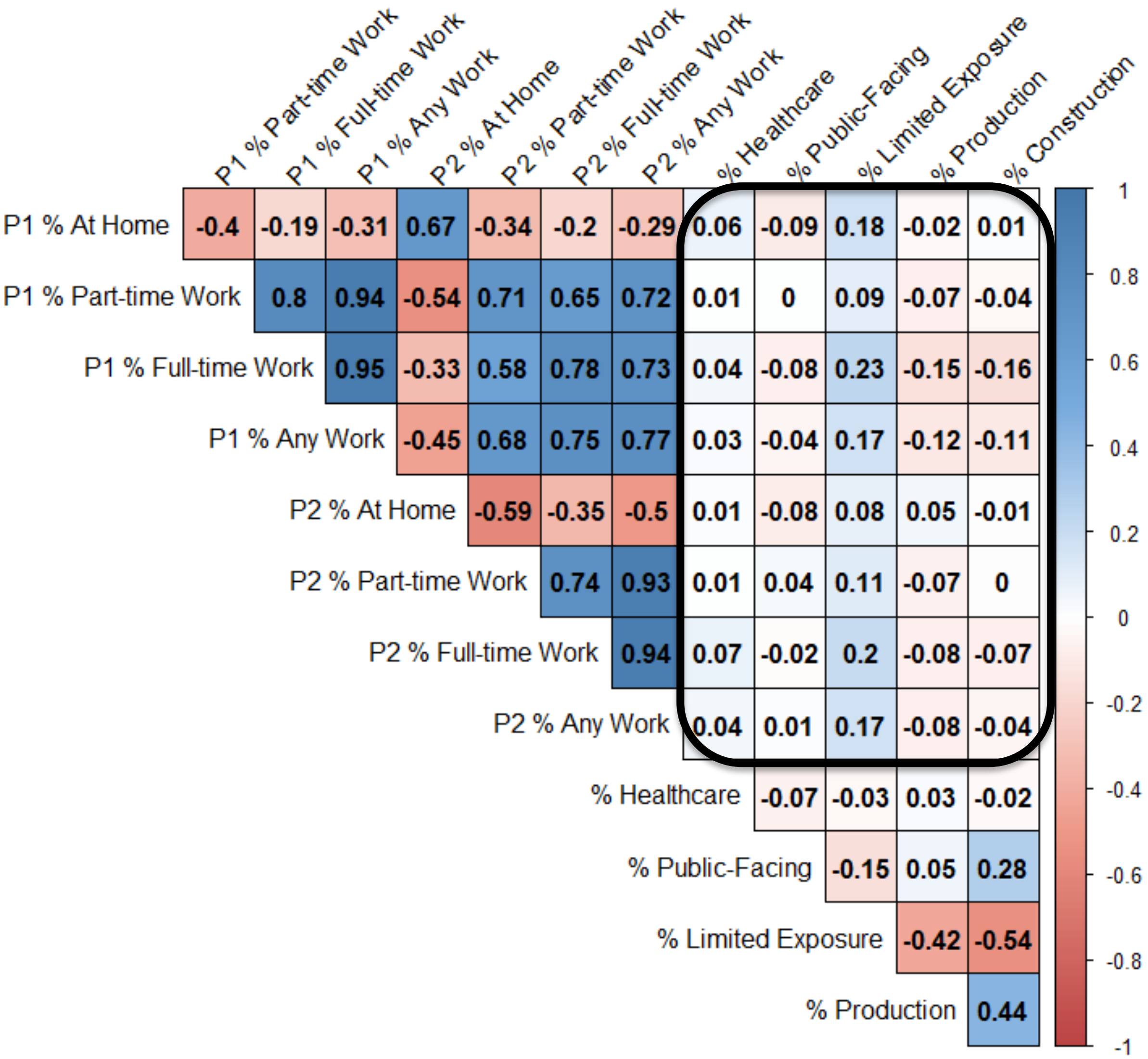

Table S2: Incidence rate ratios (IRR) and 95% confidence intervals for census tract-level factors included in regression models for non-institutional COVID-19 cases and deaths in Massachusetts by phase of the pandemic.

Note: Bold values indicate significance at 0.05 level.

	COVID-19 Non-Institutional Cases: Incidence Rate Ratios (95% CI)		COVID-19 Non-Institutional Deaths: Incidence Rate Ratios (95% CI)	
	Phase 1	Phase 2	Phase 1	Phase 2
Construction/Transportation: High	**1.32 (1.22, 1.42)**	**1.19 (1.13, 1.25)**	1.21 (1.00, 1.47)	**1.16 (1.01, 1.33)**
Construction/Transportation: Medium	**1.22 (1.14, 1.30)**	**1.16 (1.11, 1.21)**	**1.20 (1.02, 1.41)**	**1.13 (1.00, 1.26)**
Production: High	**1.23 (1.15, 1.33)**	**1.18 (1.12, 1.24)**	0.90 (0.75, 1.09)	1.10 (0.97, 1.25)
Production: Medium	**1.16 (1.09, 1.23)**	**1.12 (1.08, 1.17)**	1.03 (0.88, 1.20)	1.02 (0.91, 1.14)
Public-facing: High	**1.14 (1.07, 1.21)**	**1.10 (1.06, 1.15)**	1.03 (0.88, 1.20)	1.04 (0.94, 1.16)
Public-facing: Medium	**1.12 (1.06, 1.19)**	**1.08 (1.03, 1.12)**	1.07 (0.92, 1.23)	1.00 (0.91, 1.11)
Healthcare: High	**1.11 (1.05, 1.18)**	**1.06 (1.02, 1.11)**	1.11 (0.96, 1.29)	1.05 (0.95, 1.17)
Healthcare: Medium	1.05 (0.99, 1.11)	**1.05 (1.01, 1.09)**	1.08 (0.93, 1.24)	1.06 (0.96, 1.17)
Limited exposure: High	**0.85 (0.78, 0.94)**	**0.83 (0.77, 0.88)**	1.03 (0.81, 1.30)	**0.75 (0.64, 0.89)**
Limited Exposure: Medium	**0.91 (0.85, 0.98)**	0.96 (0.91, 1.01)	0.96 (0.80, 1.14)	0.90 (0.80, 1.01)
% Latinx	**1.38 (1.33, 1.44)**	**1.17 (1.14, 1.20)**	**1.35 (1.23, 1.49)**	**1.12 (1.05, 1.20)**
% Black	**1.26 (1.22, 1.29)**	1.01 (0.96, 1.03)	**1.28 (1.19, 1.37)**	**1.07 (1.01, 1.12)**
% AIAN	1.02 (1.00, 1.05)	1.01 (0.99, 1.02)	1.00 (0.94, 1.06)	0.99 (0.95, 1.03)
% Age < 20	**0.91 (0.88, 0.94)**	**0.95 (0.93, 0.97)**	**0.81 (0.74, 0.88)**	**0.91 (0.85, 0.97)**
% Age > 80	**1.07 (1.04, 1.10)**	1.00 (0.98, 1.02)	**1.34 (1.25, 1.43)**	**1.26 (1.20, 1.32)**
% Undergrad	**0.89 (0.86, 0.92)**	**0.95 (0.93, 0.97)**	0.92 (0.83, 1.02)	**0.87 (0.81, 0.94)**
% Uninsured	**1.08 (1.05, 1.11)**	**1.04 (1.02, 1.07)**	0.98 (0.91, 1.05)	1.01 (0.96, 1.07)
% Under federal poverty line	**1.05 (1.01, 1.09)**	**1.03 (1.01, 1.06)**	0.95 (0.87, 1.05)	**1.09 (1.03, 1.17)**
Household median income	0.97 (0.94, 1.00)	**0.96 (0.94, 0.98)**	**0.83 (0.74, 0.93)**	**0.88 (0.81, 0.95)**
% Crowding (1.5+/room)	1.01 (0.98, 1.04)	0.99 (0.97, 1.01)	1.03 (0.96, 1.10)	0.98 (0.94, 1.03)
Housing density (units/mi^2)	0.97 (0.93, 1.00)	**0.96 (0.94, 0.99)**	0.95 (0.87, 1.05)	**0.91 (0.84, 0.99)**

Table S3: Incidence rates (% of group population) in census tracts across tertiles of occupational group percentages over both Phases of the study period.

Non-institutional cases

Occupational group	Low percentile		Medium percentile		High percentile	
	Phase 1	Phase 2	Phase 1	Phase 2	Phase 1	Phase 2
Construction/ Transportation	0.68	4.12	1.10	5.97	1.74	7.27
Production	0.81	4.27	1.12	5.70	1.57	7.35
Public-facing	0.98	5.11	1.16	5.92	1.33	6.19
Healthcare	1.16	5.59	1.09	5.80	1.22	5.85
Limited Exposure	1.88	7.66	0.91	5.54	0.78	4.28

Non-institutional deaths

Occupational group	Low percentile		Medium percentile		High percentile	
	Phase 1	Phase 2	Phase 1	Phase 2	Phase 1	Phase 2
Construction/ Transportation	0.032	0.039	0.044	0.056	0.043	0.061
Production	0.036	0.040	0.041	0.050	0.042	0.065
Public-facing	0.038	0.050	0.040	0.052	0.040	0.052
Healthcare	0.037	0.048	0.044	0.054	0.038	0.053
Limited Exposure	0.051	0.068	0.035	0.051	0.034	0.037

Table S4: Incidence rate ratios (IRR) and 95% confidence intervals for census tract-level factors in regression models including interaction terms (% Black x essential worker categories and % Latinx x essential worker categories) for non-institutional COVID-19 cases and deaths in Massachusetts by phase of the pandemic.

Note: Bold values indicate significance at 0.05 level.

	COVID-19 Non-Institutional Cases: Incidence Rate Ratios (95% CI)		COVID-19 Non-Institutional Deaths: Incidence Rate Ratios (95% CI)	
	Phase 1	Phase 2	Phase 1	Phase 2
Construction/Transportation: High	**1.31 (1.21, 1.41)**	**1.19 (1.12, 1.25)**	1.21 (0.99, 1.46)	1.14 (0.99, 1.30)
Construction/Transportation: Medium	**1.23 (1.15, 1.31)**	**1.16 (1.11, 1.21)**	**1.21 (1.02, 1.42)**	1.11 (0.98, 1.24)
Production: High	**1.21 (1.13, 1.31)**	**1.18 (1.12, 1.25)**	0.85 (0.71, 1.03)	1.09 (0.95, 1.24)
Production: Medium	**1.15 (1.08, 1.22)**	**1.13 (1.08, 1.18)**	0.98 (0.83, 1.15)	1.00 (0.89, 1.12)
Public-facing: High	**1.13 (1.07, 1.20)**	**1.10 (1.06, 1.15)**	1.01 (0.86, 1.18)	1.05 (0.94, 1.17)
Public-facing: Medium	**1.12 (1.05, 1.18)**	**1.08 (1.03, 1.12)**	1.06 (0.91, 1.22)	1.01 (0.91, 1.11)
Healthcare: High	**1.11 (1.04, 1.17)**	**1.06 (1.02, 1.11)**	1.10 (0.95, 1.28)	1.05 (0.94, 1.17)
Healthcare: Medium	1.05 (1.00, 1.11)	**1.05 (1.01, 1.09)**	1.07 (0.93, 1.24)	1.05 (0.95, 1.16)
Limited exposure: High	**0.87 (0.79, 0.96)**	**0.78 (0.73, 0.83)**	1.04 (0.81, 1.34)	**0.71 (0.59, 0.85)**
Limited Exposure: Medium	**0.92 (0.86, 0.99)**	0.98 (0.93, 1.03)	0.93 (0.78, 1.12)	**0.88 (0.78, 0.99)**
% Latinx	**1.39 (1.24, 1.56)**	**1.21 (1.12, 1.31)**	**1.47 (1.12, 1.94)**	**1.31 (1.08, 1.60)**
% Black	**1.25 (1.14, 1.37)**	1.03 (0.97, 1.11)	1.14 (0.92, 1.42)	1.03 (0.87, 1.23)
% AIAN	1.02 (1.00, 1.05)	1.01 (0.99, 1.02)	0.99 (0.94, 1.06)	0.99 (0.96, 1.04)
% Age < 20	**0.92 (0.89, 0.95)**	**0.95 (0.92, 0.97)**	**0.82 (0.75, 0.90)**	**0.92 (0.86, 0.98)**
% Age > 80	**1.08 (1.05, 1.11)**	1.01 (0.99, 1.03)	**1.33 (1.24, 1.43)**	**1.27 (1.21, 1.33)**
% Undergrad	**0.88 (0.85, 0.91)**	**0.95 (0.93, 0.98)**	0.91 (0.82, 1.01)	**0.87 (0.80, 0.94)**
% Uninsured	**1.07 (1.04, 1.10)**	**1.04 (1.02, 1.07)**	0.96 (0.89, 1.04)	1.02 (0.97, 1.08)
% Under federal poverty line	**1.06 (1.02, 1.10)**	1.02 (0.99, 1.05)	0.97 (0.88, 1.07)	1.07 (1.00, 1.15)
HMI	0.97 (0.95, 1.01)	**0.96 (0.95, 0.98)**	**0.82 (0.73, 0.92)**	**0.88 (0.81, 0.95)**
% Crowding (1.5+/room)	1.00 (0.98, 1.03)	0.99 (0.98, 1.01)	1.02 (0.95, 1.09)	0.98 (0.94, 1.03)
HUD (units/mi^2)	**0.96 (0.93, 1.00)**	**0.97 (0.95, 0.99)**	0.96 (0.87, 1.05)	0.93 (0.85, 1.00)
% Latinx x Construction/Transportation: High	1.02 (0.94, 1.12)	1.01 (0.95, 1.08)	0.94 (0.77, 1.15)	0.88 (0.77, 1.02)
% Latinx x Construction/Transportation: Medium	0.97 (0.90, 1.06)	1.00 (0.94, 1.06)	0.90 (0.75, 1.09)	0.91 (0.79, 1.04)
% Latinx x Production: High	0.95 (0.87, 1.05)	0.98 (0.92, 1.04)	0.96 (0.77, 1.19)	0.96 (0.82, 1.12)
% Latinx x Production: Medium	0.99 (0.90, 1.08)	1.00 (0.93, 1.06)	0.87 (0.70, 1.08)	0.90 (0.77, 1.05)

% Latinx x Public-facing: High	**1.08 (1.01, 1.15)**	0.97 (0.92, 1.01)	1.13 (0.97, 1.32)	1.01 (0.91, 1.12)
% Latinx x Public-facing: Medium	1.05 (0.99, 1.11)	0.99 (0.95, 1.03)	1.02 (0.89, 1.17)	0.97 (0.88, 1.06)
% Latinx x Healthcare: High	0.95 (0.90, 1.01)	0.98 (0.94, 1.03)	0.96 (0.84, 1.11)	1.09 (0.99, 1.20)
% Latinx x Healthcare: Medium	**0.93 (0.88, 0.99)**	0.98 (0.95, 1.02)	1.02 (0.89, 1.17)	1.00 (0.91, 1.10)
% Latinx x Limited exposure: High	1.04 (0.91, 1.18)	**0.84 (0.77, 0.91)**	0.89 (0.64, 1.22)	0.78 (0.59, 1.02)
% Latinx x Limited Exposure: Medium	1.03 (0.94, 1.14)	1.04 (0.97, 1.11)	0.92 (0.72, 1.17)	0.93 (0.79, 1.10)
% Black x Construction: High	0.94 (0.87, 1.02)	0.96 (0.91, 1.02)	1.03 (0.85, 1.24)	1.06 (0.91, 1.23)
% Black x Construction: Medium	0.96 (0.89, 1.04)	0.97 (0.92, 1.03)	1.04 (0.86, 1.26)	1.04 (0.90, 1.22)
% Black x Production: High	**1.18 (1.10, 1.26)**	**1.07 (1.02, 1.12)**	**1.18 (1.00, 1.38)**	1.06 (0.94, 1.19)
% Black x Production: Medium	1.04 (0.98, 1.10)	1.02 (0.97, 1.06)	**1.16 (1.00, 1.33)**	1.08 (0.97, 1.20)
% Black x Public-facing: High	**0.91 (0.86, 0.96)**	**0.95 (0.91, 0.99)**	0.98 (0.86, 1.12)	0.95 (0.86, 1.05)
% Black x Public-facing: Medium	**0.92 (0.86, 0.98)**	0.97 (0.92, 1.01)	0.95 (0.83, 1.10)	0.98 (0.88, 1.08)
% Black x Healthcare: High	1.04 (0.98, 1.10)	0.99 (0.95, 1.03)	0.98 (0.85, 1.12)	0.93 (0.84, 1.03)
% Black x Healthcare: Medium	1.03 (0.96, 1.10)	1.02 (0.97, 1.08)	0.90 (0.76, 1.07)	0.88 (0.78, 1.00)
% Black x Limited exposure: High	**1.15 (1.03, 1.28)**	1.05 (0.97, 1.13)	**1.44 (1.11, 1.87)**	1.21 (0.98, 1.50)
% Black x Limited Exposure: Medium	**1.12 (1.04, 1.21)**	1.05 (0.99, 1.10)	1.09 (0.92, 1.30)	1.10 (0.97, 1.24)
