# Supplementary material for: Evaluating COVID-19 Risk to Essential Workers by Occupational Group: A Case Study in Massachusetts
Source: J Community Health. 2023 Jul 29;49(1):91–9. doi: 10.1007/s10900-023-01249-x (PMC10823035; doi:10.1007/s10900-023-01249-x)
Supplement: Supplementary file 1 [file 10900_2023_1249_MOESM1_ESM.docx]

**Evaluating COVID-19 risk to essential workers by occupational group: A case study in Massachusetts**

Journal of Community Health

Beth M. Haley^1^, Prasad Patil^2^, Jonathan I. Levy^1^, Keith R. Spangler^1^, Koen F. Tieskens^1^, Fei Carnes^1^, Xiaojing Peng^2^, R. Monina Klevens^3^, T. Scott Troppy^3^, M. Patricia Fabian^1^, Kevin J. Lane^1^, Jessica H. Leibler^1^

1. Department of Environmental Health, Boston University School of Public Health, Boston, MA, USA

2. Department of Biostatistics, Boston University School of Public Health, Boston, MA, USA

3. Bureau of Infectious Disease and Laboratory Sciences, Massachusetts Department of Public Health, Boston, MA, USA

Corresponding author:

Jessica H. Leibler

Department of Environmental Health

Boston University School of Public Health

715 Albany St.

Boston, MA, 02118

[jleibler@bu.edu](mailto:jleibler@bu.edu)

**SUPPLEMENTARY MATERIALS:**

*Table S1: Descriptive statistics of non-institutional COVID-19 cases and deaths, and average census tract characteristics in Massachusetts, USA*^*a*^*.*

|  | **Phase 1 (March 2020 – June 2020)** |  | **Phase 2 (September 2020 – February 2021)** |  |
| --- | --- | --- | --- | --- |
|  | Cases | Deaths | Cases | Deaths |
| Number of individuals^b^ | 79,349 | 2696 | 393,541 | 3535 |
| **Mean Tract-Level Characteristics** |  |  |  |  |
| Variable Name | Description |  |  | Mean (Std. Dev.) |
| % Black | Percent of the population that identifies as Black or African American |  |  | 7.94% (13.19%) |
| % Latinx | Percent of the population that identifies as Latino or Hispanic |  |  | 12.66% (16.83%) |
| % AIAN | Percent of the population that identifies as American Indian or Alaska Native |  |  | 0.23% (0.64%) |
| % Age < 20 | Percent of the population younger than 20 years old |  |  | 22.64% (6.73%) |
| % Age > 80 | Percent of the population over 80 years old |  |  | 4.23% (2.72%) |
| % Undergrad | Percent of the population enrolled as undergraduate students |  |  | 6.48% (10.65%) |
| % Uninsured | Percent of the population without health insurance |  |  | 2.47% (2.12%) |
| % Under federal poverty line | Percent of the population living in poverty |  |  | 10.80% (9.84%) |
| % Crowding | Percent of the occupied units with more than 1.5 people per room |  |  | 0.71% (1.23%)^c^ |
| HMI | Household median income (USD) |  |  | $85,878.79 ($37,892.54)^d^ |
| HUD | Housing unit density (units/mi^2^) |  |  | 3093.25 (5058.34) |

^a ^Only census tracts with population greater than 0 included in the analysis (n=1462).

^b ^Reflects the number of cases and deaths that were successfully geocoded to a census tract (total number of individuals excluded due to lack of geocoding is 1360 [0.27%]).

^c^ Three census tracts omitted because number of occupied units was 0.

^d^ Twelve census tracts omitted because household median income data missing.

*Figure S1: Correlation matrix of essential worker groups (percent of total tract population) and metrics of mobility from SafeGraph’s Social Distancing Metrics dataset. Correlations between essential worker categories and mobility metrics circled for emphasis.*

*Table S2: Incidence rate ratios (IRR) and 95% confidence intervals for census tract-level factors included in regression models for non-institutional COVID-19 cases and deaths in Massachusetts by phase of the pandemic.*

*Note: Bold values indicate significance at 0.05 level.* 

|  | COVID-19 Non-Institutional Cases: Incidence Rate Ratios (95% CI) |  | COVID-19 Non-Institutional Deaths: Incidence Rate Ratios (95% CI) |  |
| --- | --- | --- | --- | --- |
|  | Phase 1 | Phase 2 | Phase 1 | Phase 2 |
| Construction/Transportation: High | **1.32 (1.22, 1.42)** | **1.19 (1.13, 1.25)** | 1.21 (1.00, 1.47) | **1.16 (1.01, 1.33)** |
| Construction/Transportation: Medium | **1.22 (1.14, 1.30)** | **1.16 (1.11, 1.21)** | **1.20 (1.02, 1.41)** | **1.13 (1.00, 1.26)** |
| Production: High | **1.23 (1.15, 1.33)** | **1.18 (1.12, 1.24)** | 0.90 (0.75, 1.09) | 1.10 (0.97, 1.25) |
| Production: Medium | **1.16 (1.09, 1.23)** | **1.12 (1.08, 1.17)** | 1.03 (0.88, 1.20) | 1.02 (0.91, 1.14) |
| Public-facing: High | **1.14 (1.07, 1.21)** | **1.10 (1.06, 1.15)** | 1.03 (0.88, 1.20) | 1.04 (0.94, 1.16) |
| Public-facing: Medium | **1.12 (1.06, 1.19)** | **1.08 (1.03, 1.12)** | 1.07 (0.92, 1.23) | 1.00 (0.91, 1.11) |
| Healthcare: High | **1.11 (1.05, 1.18)** | **1.06 (1.02, 1.11)** | 1.11 (0.96, 1.29) | 1.05 (0.95, 1.17) |
| Healthcare: Medium | 1.05 (0.99, 1.11) | **1.05 (1.01, 1.09)** | 1.08 (0.93, 1.24) | 1.06 (0.96, 1.17) |
| Limited exposure: High | **0.85 (0.78, 0.94)** | **0.83 (0.77, 0.88)** | 1.03 (0.81, 1.30) | **0.75 (0.64, 0.89)** |
| Limited Exposure: Medium | **0.91 (0.85, 0.98)** | 0.96 (0.91, 1.01) | 0.96 (0.80, 1.14) | 0.90 (0.80, 1.01) |
| % Latinx | **1.38 (1.33, 1.44)** | **1.17 (1.14, 1.20)** | **1.35 (1.23, 1.49)** | **1.12 (1.05, 1.20)** |
| % Black | **1.26 (1.22, 1.29)** | 1.01 (0.96, 1.03) | **1.28 (1.19, 1.37)** | **1.07 (1.01, 1.12)** |
| % AIAN | 1.02 (1.00, 1.05) | 1.01 (0.99, 1.02) | 1.00 (0.94, 1.06) | 0.99 (0.95, 1.03) |
| % Age < 20 | **0.91 (0.88, 0.94)** | **0.95 (0.93, 0.97)** | **0.81 (0.74, 0.88)** | **0.91 (0.85, 0.97)** |
| % Age > 80 | **1.07 (1.04, 1.10)** | 1.00 (0.98, 1.02) | **1.34 (1.25, 1.43)** | **1.26 (1.20, 1.32)** |
| % Undergrad | **0.89 (0.86, 0.92)** | **0.95 (0.93, 0.97)** | 0.92 (0.83, 1.02) | **0.87 (0.81, 0.94)** |
| % Uninsured | **1.08 (1.05, 1.11)** | **1.04 (1.02, 1.07)** | 0.98 (0.91, 1.05) | 1.01 (0.96, 1.07) |
| % Under federal poverty line | **1.05 (1.01, 1.09)** | **1.03 (1.01, 1.06)** | 0.95 (0.87, 1.05) | **1.09 (1.03, 1.17)** |
| Household median income | 0.97 (0.94, 1.00) | **0.96 (0.94, 0.98)** | **0.83 (0.74, 0.93)** | **0.88 (0.81, 0.95)** |
| % Crowding (1.5+/room) | 1.01 (0.98, 1.04) | 0.99 (0.97, 1.01) | 1.03 (0.96, 1.10) | 0.98 (0.94, 1.03) |
| Housing density (units/mi^2^) | 0.97 (0.93, 1.00) | **0.96 (0.94, 0.99)** | 0.95 (0.87, 1.05) | **0.91 (0.84, 0.99)** |

*Table S3: Incidence rates (% of group population) in census tracts across tertiles of occupational group percentages over both Phases of the study period.*

*Non-institutional cases*

| Occupational group | Low percentile |  | Medium percentile |  | High percentile |  |
| --- | --- | --- | --- | --- | --- | --- |
|  | Phase 1 | Phase 2 | Phase 1 | Phase 2 | Phase 1 | Phase 2 |
| Construction/ Transportation | 0.68 | 4.12 | 1.10 | 5.97 | 1.74 | 7.27 |
| Production | 0.81 | 4.27 | 1.12 | 5.70 | 1.57 | 7.35 |
| Public-facing | 0.98 | 5.11 | 1.16 | 5.92 | 1.33 | 6.19 |
| Healthcare | 1.16 | 5.59 | 1.09 | 5.80 | 1.22 | 5.85 |
| Limited Exposure | 1.88 | 7.66 | 0.91 | 5.54 | 0.78 | 4.28 |

*Non-institutional deaths*

| Occupational group | Low percentile |  | Medium percentile |  | High percentile |  |
| --- | --- | --- | --- | --- | --- | --- |
|  | Phase 1 | Phase 2 | Phase 1 | Phase 2 | Phase 1 | Phase 2 |
| Construction/ Transportation | 0.032 | 0.039 | 0.044 | 0.056 | 0.043 | 0.061 |
| Production | 0.036 | 0.040 | 0.041 | 0.050 | 0.042 | 0.065 |
| Public-facing | 0.038 | 0.050 | 0.040 | 0.052 | 0.040 | 0.052 |
| Healthcare | 0.037 | 0.048 | 0.044 | 0.054 | 0.038 | 0.053 |
| Limited Exposure | 0.051 | 0.068 | 0.035 | 0.051 | 0.034 | 0.037 |

*Table S4: Incidence rate ratios (IRR) and 95% confidence intervals for census tract-level factors in regression models including interaction terms (% Black x essential worker categories and % Latinx x essential worker categories) for non-institutional COVID-19 cases and deaths in Massachusetts by phase of the pandemic.*

*Note: Bold values indicate significance at 0.05 level.*

|  | COVID-19 Non-Institutional Cases: Incidence Rate Ratios (95% CI) |  | COVID-19 Non-Institutional Deaths: Incidence Rate Ratios (95% CI) |  |
| --- | --- | --- | --- | --- |
|  | Phase 1 | Phase 2 | Phase 1 | Phase 2 |
| Construction/Transportation: High | **1.31 (1.21, 1.41)** | **1.19 (1.12, 1.25)** | 1.21 (0.99, 1.46) | 1.14 (0.99, 1.30) |
| Construction/Transportation: Medium | **1.23 (1.15, 1.31)** | **1.16 (1.11, 1.21)** | **1.21 (1.02, 1.42)** | 1.11 (0.98, 1.24) |
| Production: High | **1.21 (1.13, 1.31)** | **1.18 (1.12, 1.25)** | 0.85 (0.71, 1.03) | 1.09 (0.95, 1.24) |
| Production: Medium | **1.15 (1.08, 1.22)** | **1.13 (1.08, 1.18)** | 0.98 (0.83, 1.15) | 1.00 (0.89, 1.12) |
| Public-facing: High | **1.13 (1.07, 1.20)** | **1.10 (1.06, 1.15)** | 1.01 (0.86, 1.18) | 1.05 (0.94, 1.17) |
| Public-facing: Medium | **1.12 (1.05, 1.18)** | **1.08 (1.03, 1.12)** | 1.06 (0.91, 1.22) | 1.01 (0.91, 1.11) |
| Healthcare: High | **1.11 (1.04, 1.17)** | **1.06 (1.02, 1.11)** | 1.10 (0.95, 1.28) | 1.05 (0.94, 1.17) |
| Healthcare: Medium | 1.05 (1.00, 1.11) | **1.05 (1.01, 1.09)** | 1.07 (0.93, 1.24) | 1.05 (0.95, 1.16) |
| Limited exposure: High | **0.87 (0.79, 0.96)** | **0.78 (0.73, 0.83)** | 1.04 (0.81, 1.34) | **0.71 (0.59, 0.85)** |
| Limited Exposure: Medium | **0.92 (0.86, 0.99)** | 0.98 (0.93, 1.03) | 0.93 (0.78, 1.12) | **0.88 (0.78, 0.99)** |
| % Latinx | **1.39 (1.24, 1.56)** | **1.21 (1.12, 1.31)** | **1.47 (1.12, 1.94)** | **1.31 (1.08, 1.60)** |
| % Black | **1.25 (1.14, 1.37)** | 1.03 (0.97, 1.11) | 1.14 (0.92, 1.42) | 1.03 (0.87, 1.23) |
| % AIAN | 1.02 (1.00, 1.05) | 1.01 (0.99, 1.02) | 0.99 (0.94, 1.06) | 0.99 (0.96, 1.04) |
| % Age < 20 | **0.92 (0.89, 0.95)** | **0.95 (0.92, 0.97)** | **0.82 (0.75, 0.90)** | **0.92 (0.86, 0.98)** |
| % Age > 80 | **1.08 (1.05, 1.11)** | 1.01 (0.99, 1.03) | **1.33 (1.24, 1.43)** | **1.27 (1.21, 1.33)** |
| % Undergrad | **0.88 (0.85, 0.91)** | **0.95 (0.93, 0.98)** | 0.91 (0.82, 1.01) | **0.87 (0.80, 0.94)** |
| % Uninsured | **1.07 (1.04, 1.10)** | **1.04 (1.02, 1.07)** | 0.96 (0.89, 1.04) | 1.02 (0.97, 1.08) |
| % Under federal poverty line | **1.06 (1.02, 1.10)** | 1.02 (0.99, 1.05) | 0.97 (0.88, 1.07) | 1.07 (1.00, 1.15) |
| HMI | 0.97 (0.95, 1.01) | **0.96 (0.95, 0.98)** | **0.82 (0.73, 0.92)** | **0.88 (0.81, 0.95)** |
| % Crowding (1.5+/room) | 1.00 (0.98, 1.03) | 0.99 (0.98, 1.01) | 1.02 (0.95, 1.09) | 0.98 (0.94, 1.03) |
| HUD (units/mi^2^) | **0.96 (0.93, 1.00)** | **0.97 (0.95, 0.99)** | 0.96 (0.87, 1.05) | 0.93 (0.85, 1.00) |
| % Latinx x Construction/Transportation: High | 1.02 (0.94, 1.12) | 1.01 (0.95, 1.08) | 0.94 (0.77, 1.15) | 0.88 (0.77, 1.02) |
| % Latinx x Construction/Transportation: Medium | 0.97 (0.90, 1.06) | 1.00 (0.94, 1.06) | 0.90 (0.75, 1.09) | 0.91 (0.79, 1.04) |
| % Latinx x Production: High | 0.95 (0.87, 1.05) | 0.98 (0.92, 1.04) | 0.96 (0.77, 1.19) | 0.96 (0.82, 1.12) |
| % Latinx x Production: Medium | 0.99 (0.90, 1.08) | 1.00 (0.93, 1.06) | 0.87 (0.70, 1.08) | 0.90 (0.77, 1.05) |
| % Latinx x Public-facing: High | **1.08 (1.01, 1.15)** | 0.97 (0.92, 1.01) | 1.13 (0.97, 1.32) | 1.01 (0.91, 1.12) |
| % Latinx x Public-facing: Medium | 1.05 (0.99, 1.11) | 0.99 (0.95, 1.03) | 1.02 (0.89, 1.17) | 0.97 (0.88, 1.06) |
| % Latinx x Healthcare: High | 0.95 (0.90, 1.01) | 0.98 (0.94, 1.03) | 0.96 (0.84, 1.11) | 1.09 (0.99, 1.20) |
| % Latinx x Healthcare: Medium | **0.93 (0.88, 0.99)** | 0.98 (0.95, 1.02) | 1.02 (0.89, 1.17) | 1.00 (0.91, 1.10) |
| % Latinx x Limited exposure: High | 1.04 (0.91, 1.18) | **0.84 (0.77, 0.91)** | 0.89 (0.64, 1.22) | 0.78 (0.59, 1.02) |
| % Latinx x Limited Exposure: Medium | 1.03 (0.94, 1.14) | 1.04 (0.97, 1.11) | 0.92 (0.72, 1.17) | 0.93 (0.79, 1.10) |
| % Black x Construction: High | 0.94 (0.87, 1.02) | 0.96 (0.91, 1.02) | 1.03 (0.85, 1.24) | 1.06 (0.91, 1.23) |
| % Black x Construction: Medium | 0.96 (0.89, 1.04) | 0.97 (0.92, 1.03) | 1.04 (0.86, 1.26) | 1.04 (0.90, 1.22) |
| % Black x Production: High | **1.18 (1.10, 1.26)** | **1.07 (1.02, 1.12)** | **1.18 (1.00, 1.38)** | 1.06 (0.94, 1.19) |
| % Black x Production: Medium | 1.04 (0.98, 1.10) | 1.02 (0.97, 1.06) | **1.16 (1.00, 1.33)** | 1.08 (0.97, 1.20) |
| % Black x Public-facing: High | **0.91 (0.86, 0.96)** | **0.95 (0.91, 0.99)** | 0.98 (0.86, 1.12) | 0.95 (0.86, 1.05) |
| % Black x Public-facing: Medium | **0.92 (0.86, 0.98)** | 0.97 (0.92, 1.01) | 0.95 (0.83, 1.10) | 0.98 (0.88, 1.08) |
| % Black x Healthcare: High | 1.04 (0.98, 1.10) | 0.99 (0.95, 1.03) | 0.98 (0.85, 1.12) | 0.93 (0.84, 1.03) |
| % Black x Healthcare: Medium | 1.03 (0.96, 1.10) | 1.02 (0.97, 1.08) | 0.90 (0.76, 1.07) | 0.88 (0.78, 1.00) |
| % Black x Limited exposure: High | **1.15 (1.03, 1.28)** | 1.05 (0.97, 1.13) | **1.44 (1.11, 1.87)** | 1.21 (0.98, 1.50) |
| % Black x Limited Exposure: Medium | **1.12 (1.04, 1.21)** | 1.05 (0.99, 1.10) | 1.09 (0.92, 1.30) | 1.10 (0.97, 1.24) |
